# Keeping pace with climate change: what is wrong with the evolutionary potential of upper thermal limits?

**DOI:** 10.1002/ece3.385

**Published:** 2012-10-13

**Authors:** Mauro Santos, Luis E Castañeda, Enrico L Rezende

**Affiliations:** 1 Departament de Genètica i de Microbiologia, Grup de Biologia Evolutiva (GBE), Universitat Autònoma de Barcelona08193, Bellaterra, Barcelona, Spain; 2 Instituto de Ciencias Ambientales y Evolutivas, Facultad de Ciencias, Universidad Austral de ChileValdivia, Chile

**Keywords:** CTmax, heating rate, knockdown resistance, metabolic rate, selection responses, thermotolerance

## Abstract

The potential of populations to evolve in response to ongoing climate change is partly conditioned by the presence of heritable genetic variation in relevant physiological traits. Recent research suggests that *Drosophila melanogaster* exhibits negligible heritability, hence little evolutionary potential in heat tolerance when measured under slow heating rates that presumably mimic conditions in nature. Here, we study the effects of directional selection for increased heat tolerance using *Drosophila* as a model system. We combine a physiological model to simulate thermal tolerance assays with multilocus models for quantitative traits. Our simulations show that, whereas the evolutionary response of the genetically determined upper thermal limit (CTmax) is independent of methodological context, the response in knockdown temperatures varies with measurement protocol and is substantially (up to 50%) lower than for CTmax. Realized heritabilities of knockdown temperature may grossly underestimate the true heritability of CTmax. For instance, assuming that the true heritability of CTmax in the base population is *h*^2^ = 0.25, realized heritabilities of knockdown temperature are around 0.08–0.16 depending on heating rate. These effects are higher in slow heating assays, suggesting that flawed methodology might explain the apparently limited evolutionary potential of cosmopolitan *D*. *melanogaster*.

## Introduction

The ability to adapt and tolerate ongoing rising temperatures depends to a large extent on organismal plasticity and the evolutionary potential of populations (Helmuth et al. 2005; Ghalambor et al. 2007; Angilletta 2009). In terrestrial ectotherms, the limited evidence suggests that many tropical and desert species live at temperatures near their upper thermal limits (Deutsch et al. 2008; Huey et al. 2009), resulting in mounting pressure to understand when evolutionary responses may counter rapid climate change and how to quantitate this evolutionary potential (Parmesan 2006; Skelly et al. 2007; Visser 2008; Hoffmann and Sgrò 2011). Current information appears to indicate that upper thermal limits are weakly correlated with latitude in terrestrial ectotherms (Addo-Bediako et al. 2000; Sunday et al. 2011), and recent studies have questioned the ecological relevance of artificial selection experiments that have successfully increased heat tolerance in *Drosophila* because flies were placed acutely at stressful temperatures or subjected to a fast heating rate (e.g., McColl et al. 1996; Gilchrist and Huey 1999), which may overestimate species tolerance limits (Chown et al. 2010; Hoffmann 2010). At slow and presumably more realistic heating rates, heat tolerance is substantially lower and its additive genetic variance, and consequently narrow-sense heritability (henceforth simply “heritability”), are almost negligible (Terblanche et al. 2007; Chown et al. 2009; Peck et al. 2009; Mitchell and Hoffmann 2010). These findings lead Mitchell and Hoffmann (2010, p. 699) to conclude (our addition between brackets): *If a highly adaptable species like D*. *melanogaster which exhibits generally high heritability estimates for all quantitative traits has problems mounting evolutionary responses* [for upper thermal limits], *where does this leave other species whose adaptive potential might be curtailed due to small population size or a history of selection?*

Even though the presence of evolutionary limits is certainly a matter of concern, it is debatable whether limited evolutionary potential ultimately accounts for many empirical results. It is becoming increasingly evident that methodology can have a greater impact on estimates of upper critical thermal limits (CT_max_, defined as “the maximum temperature that an organism might potentially tolerate given its physiological condition in the absence of any other hazard;” Santos et al. 2011) than the evolutionary or acclimatory responses that researchers aim to study (Lutterschmidt and Hutchinson 1997; Terblanche et al. 2007; Chown et al. 2009; Rezende et al. 2011; Santos et al. 2011; Ribeiro et al. 2012). This raises the possibility that numerous reports on evolutionary limits might ultimately reflect measurement artifacts because the magnitude of a thermal challenge during an assay depends on both the temperature and the duration of exposure to this temperature (Hochachka and Somero 2002, p. 331), and an organism's physiology as well as its probability to survive any given thermal challenge vary in time. Taking these issues into account, we have recently developed a theoretical framework that adequately reproduces the impact of methodological protocol on empirical measurements of CT_max_ in *D*. *melanogaster* and explains why measurements of CT_max_ obtained with different methods are often uncorrelated or exhibit contrasting heritability estimates (Santos et al. 2011). Here, we study the effects of directional selection for increasing heat tolerance with a theoretical approach that combines this framework with multilocus models for quantitative traits.

## Background

Rezende et al. (2011) postulated that CT_max_ can change due to acclimation and resource depletion (or fatigue) during the course of a ramping assay, in which temperature increases from an initial temperature *T*_0_ at a rate Δ*T* (°C /min) until individuals succumb to heat stress. The total amount of time under heat stress increases in slow ramping assays, which lowers CT_max_ as resources are depleted and explains why heat tolerance is often positively correlated with Δ*T* (Elliott et al. 1994; Mora and Maya 2006; Terblanche et al. 2007; Chown et al. 2009; Peck et al. 2009). Rezende et al. (2011) also demonstrated that ramping rates affect the additive genetic and residual variances of CT_max_ in opposite directions, with slow ramping rates decreasing the genetic variance, but increasing the residual variance. However, in their model, the residual variance arises from individual differences in metabolism, which consume energy and water resources at rates that are unrelated to genetic differences in CT_max_.

The theoretical approach in Rezende et al. (2011) is a simplified account of what happens in heat resistance assays, and was recently expanded by incorporating a survival probability function that varies with temperature (Santos et al. 2011). In this model, knockdown temperatures involve a deterministic component, as in Rezende et al. (2011), and a stochastic component that reflects the time-dependent cumulative probability of collapse as temperature approaches CT_max_ (see Appendix S1). As CT_max_ corresponds by definition to the upper physiological limit, knockdown temperatures will always be biased downwards with respect to this parameter, and this is the primary reason why empirical knockdown temperatures should not be equated with CT_max_. The key ingredients of Santos et al.'s (2011) model are encapsulated in the following equation (eqn 10 in their paper):


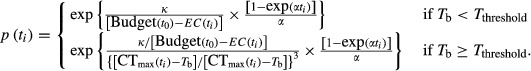


where 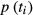 is the probability of any given individual surviving to time *t*_*i*_ (the time interval is 1 min) as a function of body temperature *T*_b_; 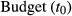 is its total reserves at *t*_0_, which is depleted during the course of the experiment at a rate determined by metabolism; 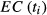 is the amount of resources consumed at time *t*_*i*_; *T*_threshold_ is the temperature at which or above which the individual is under thermal stress; and *κ* and *α* are constants. The model can accurately replicate survival times when flies are assayed for desiccation resistance (*T*_b_ < *T*_threshold_; Fig. 1 in Santos et al. 2011) or are subjected to different types of thermal stress (*T*_b_ ≥ *T*_threshold_; Fig. 3 in Santos et al. 2011).

**Figure 1:**
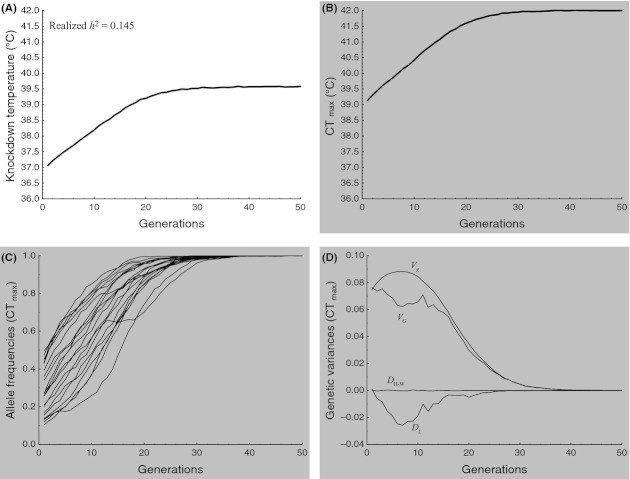
Sample numerical results from simulation model 1 assuming additive and equal allele effects for CT_max_. The census size at each generation was *N* = 5, 000 flies. They were subjected to 50 generations of up-selection for knockdown temperature (the top 20% of each sex was retained) using a fast ramping protocol with *T*_0_ = 28°C and Δ*T* = 0.5°C/min. CT_max_ was controlled by 20 diallelic loci on the same chromosome, with recombination frequency between adjacent loci *r* = 0.25 in females. Allelic frequencies in the base population ranged from p = 0.1 to p = 0.5 for alleles ^′^1^′^, which all have additive effects 0.1°C. Heritability of CT_max_ was *h*^2^ = 0.25 in the base population. Panel A plots the increase in knockdown temperature and its realized heritability, estimated by regressing the response to selection against the cumulated selection differential over the first 12 generations of selection. Panel B plots the increase in CT_max_, which was 20% higher relative to the increase in knockdown temperature. Panel C plots the frequency changes of alleles ^′^1^′^ increasing CT_max_, which eventually reached fixation. Panel D plots the total genotypic variance *V*_*G*_ together with its causal components. The genic variance component *V*_*g*_ initially increased (approximately up to generation 8) as a consequence of the changes in allele frequencies, but the genotypic variance *V*_*G*_ steadily decreased because of linkage disequilibrium (*D*_L_). Plots for CT_max_, allele frequencies, and variance components are framed in shadow because their responses to selection for knockdown temperature are hidden to the experimentalist. For results of a similar model relaxing the assumptions of equal allelic effects and strict additivity, see Appendix S3.

Whether those individuals with the highest knockdown resistance correspond to those with the highest CT_max_ will depend on the amount of noise introduced by stochasticity, which is expected to be greater in heat tolerance assays that use slow heating rates (Santos et al. 2011) that are presumably “ecologically realistic” (Chown et al. 2009; Mitchell and Hoffmann 2010; Overgaard et al. 2011a,b; but see Rezende and Santos 2012). Therefore, it remains unclear how stochasticity might affect both the selection differential during selection for increased heat tolerance and/or the power to detect any eventual evolutionary response. Here, we employ computer simulations to show that selection responses and realized heritabilities depend on methodological context for knockdown temperature (i.e., the estimator), but are essentially context-independent for CT_max_ (i.e., the parameter researchers attempt to estimate). As knockdown temperature involves a substantial amount of noise due to stochasticity, some ramping protocols may misleadingly suggest low evolutionary potential in CT_max_ when genetic adaptation has in fact taken place.

## Computer Simulations

We used computer simulations that mimic artificial selection experiments for increasing knockdown temperature in the model species *D*. *melanogaster*, employing the “Gompertz” code provided in Santos et al. (2011) to compute knockdown temperatures. The metric trait under selection was heat tolerance, measured as knockdown temperature in a ramping protocol. The target variable knockdown temperature presumably estimates the underlying parameter CT_max_, as discussed in Santos et al. (2011), which is the polygenic character that we simulate. We assumed that short-term acclimation responses (“hardening”) did not occur during the thermal tolerance assays, which is an important caveat to keep in mind in experiments.

In the initial model, CT_max_ was determined by an arbitrary number of autosomal diallelic loci with purely additive effects for simplicity (model 1 below). Tolerance to high temperatures in large outbred populations of *D*. *melanogaster* is known to be a polygenic character, presumably affected by hundreds of genes on all chromosomes (Cavicchi et al. 1995; Loeschcke et al. 1997; Sørensen et al. 2005). However, one or a few candidate genes seem to explain much of the quantitative variation that we see in nature for heat knockdown temperature (Gilchrist and Huey 1999; Rand et al. 2010), which suggests that the distribution of allele effects affecting the quantitative variability in heat tolerance can have an exponential or geometric shape with increasingly fewer genes of progressively larger effects (Shrimpton and Robertson 1988; Orr 1999; Hayes and Goddard 2001; but see Rockman 2012). As allele frequency changes during selection are highly dependent on allele frequencies and the distribution of allele effects, we also investigated the gamma distribution with shape parameter one-half and scale parameter one for the distribution of allele effects.

Next, we incorporated additional genetic variation in metabolic rates (MR) to analyze how the selection protocol might impact correlated responses on this trait that may, in turn, affect selection responses on heat tolerance (model 2). Water depletion during a thermal tolerance assay increases with metabolism (Rezende et al. 2011; see above), which is a serious concern in long assays that use slow heating rates because water content has a significant impact on heat tolerance (Maynard Smith 1957; Levins 1969; Parsons 1980; Huey et al. 1992; Block et al. 1994). In our model, variation in MR affects the rate at which resources are depleted and, consequently, 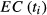 (see above). Recent debates emphasize the need to use ecologically relevant slow heating rates when extrapolating laboratory estimates of heat tolerance to field conditions (Terblanche et al. 2007; Chown et al. 2009, 2010; Hoffmann 2010; Mitchell and Hoffmann 2010; Sgrò et al. 2010). In this case, we predict a correlated response in decreasing MR when selecting flies for increased knockdown temperature.

### Model 1: Genetic variation for CT_max_

The genotypic value for CT_max_ had maximum range from a lower 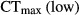 to an upper 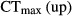 defined limits, fixed without loss of generality at 38°C and 42°C, respectively. We assumed CT_max_ to be determined by *ℓ* autosomal loci with two alleles each, ^′^1^′^ and ^′^0^′^ contributing *a* = (42–38)/2ℓ°C and 0°C, respectively; that is, we modeled purely additive effects with each locus contributing equally to CT_max_ (see Feder 1996). The genotypic value of each individual was thus 38°C plus *a* times the number of ^′^1^′^ alleles. Following Bulmer (1976), the total genotypic variance *V*_*G*_ in this model, calculated from the distribution of CT_max_ genotypic values, can be conveniently partitioned into its three causal components:


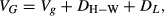


where *V*_*g*_ is the genic variance computed from the observed allele frequencies; *D*_H-W_ is the variance arising from deviations of perfect Hardy–Weinberg proportions at each locus; and *D*_L_ is the variance due to deviations of linkage equilibrium among loci. Expressions for these variances are as follows (after Bulmer 1976):


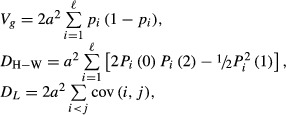


where *p*_*i*_ is the frequency of allele ^′^1^′^ at the *i*th locus; 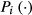 is the frequency of genotypes with 0, 1, and 2 alleles ^′^1^′^ at the *i*th locus; and 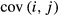 is the covariance between the number of alleles ^′^1^′^ at loci *i* and *j* (in the simulations *D*_*L*_ is computed as *D*_*L*_ = *V*_*G*_ − *V*_*g*_ − *D*_H−W_). Assuming random mating *D*_H−W_ varies around zero due to sampling fluctuations, but *D*_*L*_ will depart from zero due to the effects of selection (Felsenstein 1965; Bulmer 1971, 1976). The *ℓ* loci controlling CT_max_ were assumed to be on the same chromosome for simplicity, and the recombination fraction between adjacent loci was zero in males (as it happens in *Drosophila*) and *r* in females with no interference. The recombination process followed the stochastic multilocus method described in Fraser and Burnell (1970). Briefly, the simulation of recombination involves the equivalent of a random walk along the length of the pair of homologous chromosomes, changing from one homologue to the other within the constraint of the probability of recombination.

The phenotypic values for CT_max_ were obtained by adding a normally distributed environmental component with zero mean and variance *V*_*e*_ to the genotypic values. Importantly, this environmental variance *V*_*e*_ is the variation in phenotype that cannot be explained by identifiable genetic differences and can be assumed to arise from uncontrolled random developmental variation among individuals. It affects the genetically determined basal CT_max_ *per se,* and therefore has nothing to do with the additional residual variation that arises from stochasticity when estimating knockdown temperatures in experiments (Santos et al. 2011; see above). In other words, *V*_*e*_ is the “real” environmental variance component that quantitative geneticists routinely introduce when modeling a metric trait (Falconer and Mackay 1996).

The census population at each generation was *N*, with an equal number of females and males. All individuals were scored for their knockdown temperatures using the “Gompertz” computer code provided in Santos et al. (2011), and directional selection was applied by retaining the top 20% individuals from each sex at each generation (simulated individuals do not become sterilized after high temperature exposure; see Discussion). These selected individuals produced 2*N* gametes that were paired at random to render the next generation. The simulations were continued for *g* generations of directional selection. All simulations for model 1 assumed an average fruit fly weighing 1 mg with constant MR of 4.2 mL O_2_/g/h at 18°C, or 0.07 *μ*LO_2_ per fly min (Berrigan and Partridge 1997). Therefore, MR does not contribute to the residual variation in CT_max_ as assumed by Rezende et al. (2011). We also assumed that its total energy budget before the heat knockdown assay was equal to 171.6 *μ*LO_2_, and *Q*_10_ = 3.5 (see Santos et al. 2011).

### Model 2: Genetic variation for CT_max_ and MR

Our second model explicitly takes into account genetic differences in MR (known to be responsive to laboratory selection in *Drosophila*; Williams et al. 1997) to analyze how correlated responses in this trait could influence selection responses on heat tolerance. We recall that mortality rates in a heat tolerance assay may be partly determined by MR because this variable determines how fast water and energy resources are depleted (eq. 1; see also Rezende et al. 2011; Santos et al. 2011). We assumed *ℓ* autosomal diallelic loci controlling MR with equal and additive effects for simplicity (see Simulation Results). The locations of all *ℓ* + *ℓ* loci for CT_max_ and MR were randomly assigned to the chromosome, and recombination frequency between adjacent loci was modeled as previously indicated.

The genotypic value for MR had maximum range from 3.4 mL O_2_/g/h to 5.6 mL O_2_/g/h at 18°C (0.057 *μ*LO_2_ per fly min and 0.093 *μ*LO_2_ per fly min, respectively). The reason for this range is that the initial frequencies of ^′^1^′^ alleles increasing MR were randomly generated from a uniform distribution between 0.1 and 0.5, resulting in an average MR in the base population before selection close to 4.2 mL O_2_/g/h at 18°C (0.07 *μ*LO_2_ per fly min) as in simulation model 1. The phenotypic values for MR were obtained by adding a normally distributed environmental component with zero mean and variance 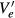 to the genotypic values. The average fruit fly also weighted 1mg and its total energy budget prior to the heat knockdown assay was 171.6 *μ*LO_2_, and *Q*_10_ = 3.5.

The metric trait under selection was heat tolerance, measured as knockdown temperature as before. Note that any correlation arisen between CT_max_ and MR during selection is not due to pleiotropy because the loci were assumed to affect each trait independently. Although linkage can be a cause of transient correlation, correlated responses in MR when up-selecting flies for knockdown temperature will be mainly caused by the “environment” (Falconer and Mackay 1996), which here means the methodology employed to score the flies (i.e., heating rate in the ramping assay).

The simulation programs were implemented in the MATLAB algebra program environment (V7; MathWorks 2005) together with the collection of tools supplied by the Statistics Toolbox. The computer code is available upon request from the corresponding author.

## Simulation Results

In all simulations, the census size was *N* = 5000 flies at each generation. Females and males were selected separately for knockdown temperature, and the top 20% of each sex was retained (i.e., 500 females and 500 males). The number of loci was *ℓ* = 20, 40 with initial frequencies of alleles ^′^1^′^ randomly generated from a uniform distribution between 0.1 and 0.5. Recombination frequencies were assumed to be *r* = 0.05, 0.15, 0.25 in females (population recombination frequency should be multiplied by one-half because *Drosophila* males lack recombination). Unless otherwise stated, the heritability of CT_max_ (and MR; simulation model 2) was assumed to be *h*^2^ = 0.25 in the base population before selection, a reasonable value for physiological traits (Mousseau and Roff 1987; Roff and Mousseau 1987).

### Model 1: Genetic variation for CT_max_

**Figure 2:**
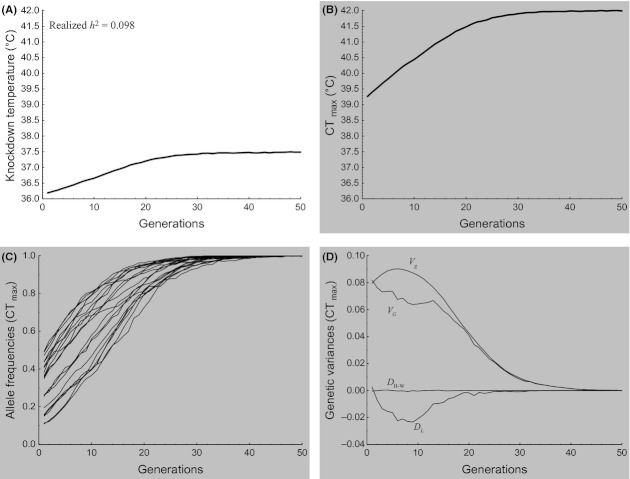
Sample numerical results from simulation model 1 using the same base population than in Fig. 1. Flies were subjected to 50 generations of up-selection for knockdown temperature (the top 20% of each sex was retained) using a slow ramping protocol with *T*_0_ = 28°C and Δ*T* = 0.06°C/min. Panel A plots the increase in knockdown temperature and its realized heritability, estimated by regressing the response to selection against the cumulated selection differential over the first 12 generations of selection. Panel B plots the increase in CT_max_, which was 2.5 times higher than the increase in knockdown temperature. Panel C plots the frequency changes of alleles ^′^1^′^ increasing CT_max_, which eventually reached fixation. Panel D plots the total genotypic variance *V*_*G*_ together with its causal components. Plots for CT_max_, allele frequencies and variance components are framed in shadow because their responses to selection for knockdown temperature are hidden to the experimentalist. For results of a similar model relaxing the assumptions of equal allelic effects and strict additivity, see Appendix S3.

Results from sample simulations with *ℓ* = 20 and *r* = 0.25 are plotted in Fig. 1A–D for flies selected with a fast ramping assay (*T*_0_ = 28°C, Δ*T* = 0.5°C/min), and in Fig. 2A–D for flies selected with a slow ramping protocol (*T*_0_ = 28°C, Δ*T* = 0.06°C/min). In both simulations, we assumed additive and equal allele effects for CT_max_. The per-locus contribution to the additive genetic variance in the base population ranged from 1.8 × 10^−3^(°C)^2^ when p = 0.1 to 5.0 × 10^−3^(°C)^2^ when p = 0.5.

With fast ramping knockdown temperature rose from approximately 37°C to 39.5°C (Δkt_fast_ = 2.5°C), the realized heritability was 0.145 (Fig. 1A). However, under slow ramping, the increase in knockdown temperature was from approximately 36.2°C to 37.4°C (Δkt_slow_ = 1.2°C) with realized heritability 0.098 (Fig. 2A). In both situations, the underlying heritability of CT_max_ (*h*^2^ ≍ 0.25) was grossly underestimated. Importantly, the response of CT_max_ to directional selection on knockdown temperature was essentially independent of the ramping conditions: it rose from an initial temperature of approximately 39°C and plateaued around the maximum attainable temperature of 42°C (ΔCT_max_ = 3°C) (Fig. 1B, 2B) once alleles increasing CT_max_ went to fixation (Fig. 1C, 2C). As expected from theory, the genotypic variance of CT_max_ was reduced by directional selection because of the generation of negative gametic linkage disequilibrium (Fig. 1D, 2D), the so-called “Bulmer effect” (Bulmer 1971).

We performed extensive computer simulations to cover a wide range of experimental conditions for all combinations of number of loci (*ℓ* = 20, 40) determining CT_max_ and recombination frequencies (*r* = 0.05, 0.15, 0.25). For each combination of parameter values, the same initial population of *N* = 5000 flies was subjected to 12 generations of up-selection for knockdown resistance under 400 different ramping protocols, after combining 20 initial temperatures (*T*_0_ ranging from 15°C to 34°C with interval 1°C) with 20 heating rates (*ΔT* ranging from 0.05 to 1°C/min with interval 0.05°C/min). Realized heritabilities for knockdown temperature, as well as the absolute increase of knockdown temperature and CT_max_ after selection, against *T*_0_ and Δ*T* are given as 3-D surface plots in Appendix S2. The numerical results provide a clear snapshot on how different ramping rates affect the response of directional selection for knockdown temperature, the realized heritabilities, and the underlying genetic responses in CT_max_. Several conclusions emerge and can be summarized as follows: (1) The efficiency of selection in changing the allele frequencies and the trait mean (knockdown temperature, CT_max_) obviously depends on the magnitude of allele effects, which decreases with an increasing number of loci *ℓ*. (2) The effectiveness of selection is slightly lower when recombination frequencies are low (*r* = 0.05) as expected by the Hill–Robertson effect (Hill and Robertson 1966; Felsenstein 1974), which establishes that the tighter is the linkage the higher is the perturbation that selection at one locus will have on other loci. (3) Realized heritabilities for knockdown temperature are in the range 0.08–0.16 and grossly underestimate the true heritability of CT_max_ (*h*^2^ ≍ 0.25), if both *T*_0_ and Δ*T* are high realized heritabilities for knockdown temperature increase by about 60% when compared with slow ramping protocols that start with an initially low or moderate *T*_0_. (4) The increase of knockdown temperature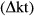 after 12 generations of selection can differ up to two or threefold according to the methodology, with Δkt being higher when both *T*_0_ and Δ*T* are high. (5) The underlying increase in CT_max_ is always higher than Δkt and more or less independent of the methodological approach, with a maximum difference across ramping protocols generally less than 20%.

Realized heritabilities for knockdown temperature will obviously change according to the underlying heritability of CT_max_. For instance, assuming *h*^2^ = 0.1 for CT_max_ in the base population simulations as those performed in Fig. 1A–D and Fig. 2A–D show that realized heritability for knockdown temperature is around 0.076 with fast ramping and 0.054 with slow ramping; and with *h*^2^ = 0.4, the corresponding values are 0.186 with fast ramping and 0.132 with slow ramping. However, the important point is that realized heritabilities for knockdown temperature will always underestimate the “true” heritability of CT_max_, and the underestimation is higher the slower the ramping rate. Needless to say, if the true heritability of CT_max_ is low, the power to detect a realized heritability for knockdown temperature significantly different from zero under “ecologically realistic” slow ramping rates will likely be very low. What our simulations emphasize is that, with certain experimental approaches, it is impossible to discriminate if low heritabilities reflect a biological phenomenon or a measurement artifact.

Our next step was to analyze to what extent the previous conclusions are robust to simplifying genetic details; namely, additive and equal allele effects for CT_max_. Assuming unequal allele effects with nonadditive contributions to CT_max_ simulations show that the previous conclusions quantitatively hold (Appendix S3).

### Model 2: Incorporating genetic variation in metabolic rates

As correlated responses in decreasing MR may occur when selecting flies for increased knockdown temperature, our final model explicitly takes into account genetic differences for both CT_max_ and MR to analyze to what extent a reduction in MR can affect our previous conclusions, and also because variation in MR introduces an additional source of residual variance when scoring flies for knockdown temperature (see above). We assumed a simple additive and equal allele effects model because our previous simulations showed that numerical results were robust to these simplifying assumptions (Appendix S3).

**Figure 3:**
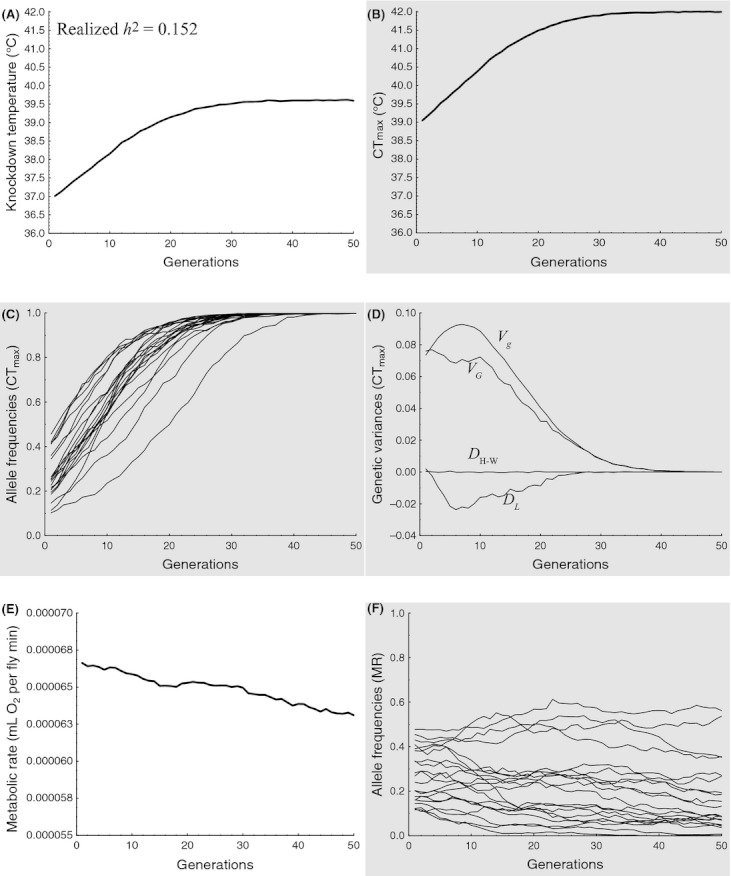
Sample numerical results from simulation model 2 assuming genetic variation for CT_max_ and MR. The census size at each generation was *N* = 5, 000 flies. They were subjected to 50 generations of up-selection for knockdown temperature (the top 20% of each sex was retained) using a fast ramping protocol with *T*_0_ = 28°C and Δ*T* = 0.5°C/min. Both CT_max_ and MR were independently controlled by 20 diallelic loci on the same chromosome, with recombination frequency between adjacent loci *r* = 0.25 in females. Allelic frequencies in the base population ranged from p = 0.1 to p = 0.5 for allele ^′^1^′^, which has additive effects 0.1°C for CT_max_ or 0.055 mLO_2_/g/h at 18°C for MR. Heritabilities of CT_max_ and MR were *h*^2^ = 0.25 in the base population. Panel A plots the increase in knockdown temperature and its realized heritability, estimated by regressing the response to selection against the cumulated selection differential over the first 12 generations of selection. Panel B plots the increase in CT_max_, which was 20% higher relative to the increase in knockdown temperature. Panel C plots the frequency changes of alleles ^′^1^′^ increasing CT_max_, which eventually reached fixation. Panel D plots the total genotypic variance *V*_*G*_ together with its causal components. The genic variance component *V*_*g*_ initially increased (approximately up to generation 8) as a consequence of the changes in allele frequencies, but the genotypic variance *V*_*G*_ steadily decreased because of linkage disequilibrium (*D*_L_). Panel E plots the correlated response in MR, which did not substantially change during selection. Panel F plots the frequencies of alleles ^′^1^′^ increasing MR, which fluctuated more or less randomly during selection. Plots for CT_max_, allele frequencies, and variance components are framed in shadow because their responses to selection for knockdown temperature are hidden to the experimentalist.

**Figure 4:**
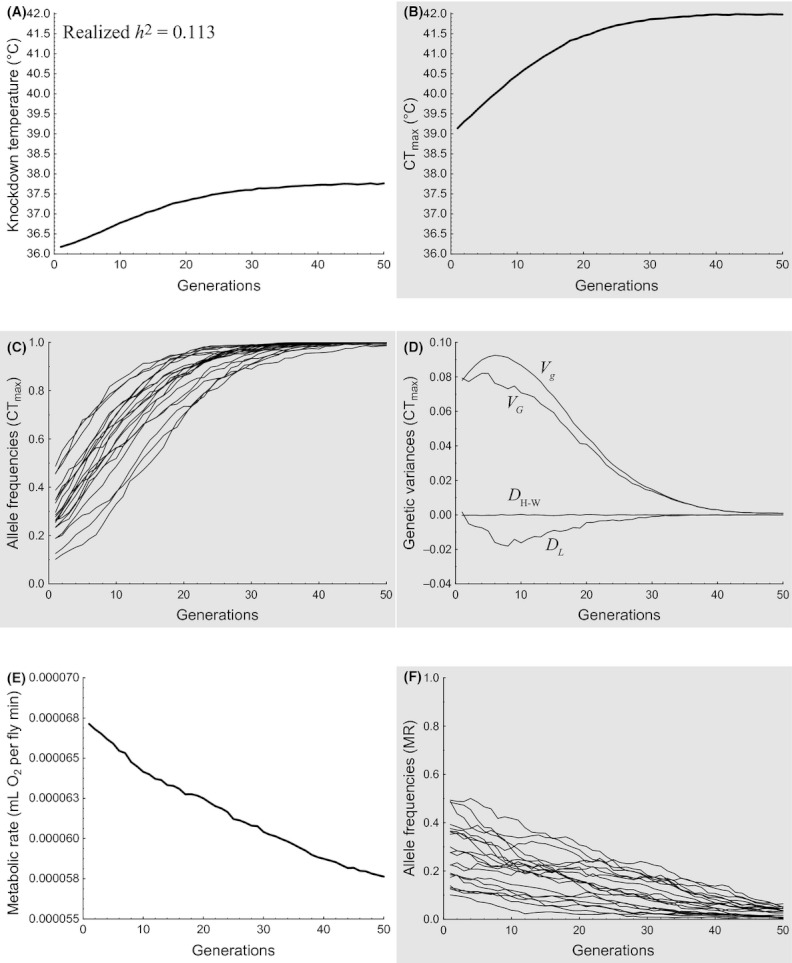
Sample numerical results from simulation model 2 using the same base population than in Fig. 3. Flies were subjected to 50 generations of up-selection for knockdown temperature (the top 20% of each sex was retained) using a slow ramping protocol with *T*_0_ = 28°C and Δ*T* = 0.06°C/min. Panel A plots the realized heritability of knockdown temperature estimated by regressing the response to selection against the cumulated selection differential over the first 12 generations of selection. Panel B plots the increase in CT_max_ alter selection, which was 115% higher relative to the increase in knockdown temperature. Panel C plots the frequency changes of alleles ^′^1^′^ increasing CT_max_, which eventually reached fixation. Panel D plots the total genotypic variance *V*_*G*_ together with its causal components. Panel E plots the correlated response in MR, which dropped by approximately 16% after selection for increasing knockdown temperature. Panel F plots the frequency changes of alleles ^′^1^′^ affecting MR, which clearly decreased during selection and were eventually lost. Plots for CT_max_, variance components, and allele frequencies are framed in shadow because their responses to selection for knockdown temperature are hidden to the experimentalist.

Results from sample simulations with *ℓ* = 20 loci for both CT_max_ and MR with *r* = 0.25 indicate that when flies are selected with a fast ramping assay (*T*_0_ = 28°C, Δ*T* = 0.5°C/min), the loci affecting MR fluctuated more or less randomly and flies' average MR did not substantially change during selection (Fig. 3E, 3F). Interestingly, *D*. *melanogaster* flies selected for knockdown temperature under a fast heating rate (*T*_0_ = 30°C, Δ*T* ≈ 0.4°C/min) did not show correlated responses in MR in the upper thermal range (Table 1 in Folk et al. 2007). Conversely, in the slow ramping protocol (*T*_0_ = 28°C, Δ*T* = 0.06°C/min), simulations show that there was a clear declining trend in the frequencies of alleles that raise MR (alleles ^′^1^′^): average MR at 18°C decreased from 0.067 *μ*LO_2_ per fly min to 0.058 *μ*LO_2_ per fly min after selection (Fig. 4E, 4F).

However, these correlated responses did not have any major impact on how different ramping rates affect the realized heritabilities for knockdown temperature (0.152 with fast ramping and 0.113 with slow ramping), although the increase in knockdown temperature after selection was approximately twice as higher with fast ramping (Δkt_fast_ = 2.5°C) than with slow ramping (Δkt_slow_ = 1.4°C) (Fig. 3A, 4A). The underlying genetic response in CT_max_ was essentially the same in both cases: it rose up to 42 °C after 30 generations (Fig.3B, 4B) once alleles increasing CT_max_ went to fixation (Fig. 3C, 4C). As before, the genotypic variance in CT_max_ was reduced by directional selection because of the generation of negative gametic linkage disequilibrium (Fig. 3D, 4D). Extensive computer simulations as those performed in Appendix S2 reinforce these conclusions (results not shown).

Finally, some simulated scenarios (Appendix S2) can be criticized for being highly unrealistic in practical terms. If the initial temperature in the ramping assay is low, say *T*_0_ ≤ 20°C, the time taken to score the flies for knockdown temperature at any generation can be higher than 3 h with slow heating rates, and acclimation effects during the assays cannot probably be ignored (Sgrò et al. 2010; Santos et al. 2011). However, for any initial temperature researchers might consider appropriate to perform the experiment (e.g., *T*_0_ = 28°C as used in the sample simulations), the previous conclusions hold.

## Discussion

A number of authors have recently questioned the standard belief that there is abundant genetic variation in any ecologically relevant trait for natural selection to act on (Blows and Hoffmann 2005). Some tropical rainforest *Drosophila* species appear to have lost heritable variation for desiccation resistance (Hoffmann et al. 2003a; Kellermann et al. 2006, 2009), an important physiological trait that might have an impact on species distributions. Selection experiments for increasing heat knockdown resistance in the cosmopolitan species *D*. *melanogaster* also suggest low but significant levels of genetic variation, with realized heritabilities around 7–12% (McColl et al. 1996; Bubli et al. 1998; Gilchrist and Huey 1999). However, flies in the experiments were acutely exposed to a high temperature or to a fast heating rate, which has raised concerns about extrapolations to field conditions because estimates under “ecologically realistic” slow heating rates suggest that the heritability of upper thermal limits is close to zero (Mitchell and Hoffmann 2010). These findings add considerable fuel to the debate on whether species will be able to persist by adapting genetically to our changing climate (Parmesan 2006; Skelly et al. 2007; Visser 2008; Chevin et al. 2010).

The speculation that the heritability of CT_max_ can change as a direct consequence of the heating rate was advanced by Chown et al. (2009). For instance, Mitchell and Hoffmann (2010) showed that the estimated heritability of heat tolerance in one Australian population (Gordonvale) of *D*. *melanogaster* dropped from *h*^2^ = 0.22 ± 0.07 when flies were placed acutely at a stressful temperature of 38°C to *h*^2^ = 0.05 ± 0.07 when assayed with a slow heating rate of 0.06°C/min. Both Chown et al. (2009) and Mitchell and Hoffmann (2010) explicitly talked about CT_max_, although what they measured was heat knockdown resistance using different methods. We have cautioned on the common misconception of equating CT_max_ with knockdown resistance (Santos et al. 2011; see above), and the present results dramatically illustrate the repercussions of our warnings. The value *h*^2^ = 0.05 ± 0.07 in Mitchell and Hoffmann (2010) is a heritability estimate of knockdown temperature under slow ramping. It is entirely consistent to simultaneously have a moderate heritability of CT_max_ and a low heritability of knockdown temperature in dynamic experiments that use a ramping rate of 0.06°C/min (Appendix S2). Their conclusion that upper thermal limits have low evolutionary potential under ecologically relevant slow heating rates is consequently incorrect. The estimate *h*^2^ = 0.22 ± 0.07 (Table 2 in Mitchell and Hoffmann 2010) is probably closer to the true heritability of CT_max_ in their population (qualitatively similar results were reported for a Melbourne population; see Mitchell and Hoffmann 2010).

### Why selection responses are context-dependent for knockdown temperature, but not for CT_max_?

The amount of noise introduced by stochasticity during a heat tolerance assay obviously lowers the correlation between knockdown temperature (estimator) and CT_max_ (parameter), and we have shown that the expected repeatability of heat tolerance can be very low (Santos et al. 2011). Actually, the limited evidence available in *Drosophila* points to a repeatability of 20% (Krebs and Loeschcke 1997; see also Santos et al. 2011). However, when selecting for increasing heat tolerance, the crucial point is to know how the selection differential applied to this phenotypic character translates to the genetic differences in the causal trait CT_max_. In our theoretical approach to model heat resistance assays (eq. 1), we explicitly incorporated the obvious assumption that survival probability steadily decreases toward zero when body temperature *T*_b_ → CT_max_. Therefore, it may happen that an individual that can tolerate, in the very best of cases, a temperature of 41°C collapses at a lower *T*_b_ of, say, 39°C because the time-dependent cumulative probability of dying at this temperature is higher than zero (importantly, the lower the heating rate in a ramping assay, the higher the probability of dying at a temperature substantially lower than CT_max_; Santos et al. 2011). But, this cannot happen the other way around. This asymmetry guarantees that the top percentile of selected individuals (20% in the simulations) for knockdown temperature includes most of the “best” individuals for CT_max_, and this is largely independent of heating rates. For instance, assume a computer-generated base population from simulation model 1 selected for knockdown temperature. A selection differential of 0.91°C (top 20%) under fast ramping translates to a selection differential of 0.67°C for CT_max_, and a selection differential of 0.39°C (top 20%) under slow ramping to a selection differential of 0.63°C for CT_max_. Although the exact magnitude of these numbers can obviously change according to the specific details of the function that describes the survival probability during a heat resistance assay, it is undoubtedly true that the previous asymmetry holds.

### Selection for increased heat tolerance

Our results suggest that CT_max_ can evolve substantially more in response to selection than is estimated by empirical measures of knockdown temperature and, most importantly, this response seems to be independent of heating rates (correlated responses in MR, on the other hand, are more pronounced under slow heating rates; cf. Figs. 3E, 4E). Although computer simulations have focused in *D*. *melanogaster* for obvious reasons, the problem is absolutely general because the estimation of upper thermal limits involves placing the individuals under stressful conditions and record the time to collapse or death. Thus, even though resource depletion during a heat resistance assay may not be a major concern for a larger organism than a *Drosophila*, stochasticity is an unavoidable source of error that downwardly bias estimates of heat tolerance and its evolutionary potential. This can be easily illustrated by setting 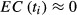 in our model (eq. 1); that is, by assuming that in the limit, the physiological condition of individuals does not decay during the heat tolerance assay. Simulations show that realized heritabilities for knockdown temperature underestimate the “true” heritability of CT_max_, and the underestimation is higher the slower the ramping rate (results not shown).

These findings have important repercussions for our understanding of the evolution of thermal tolerance for two reasons. First, they suggest that the methodology employed can seriously underestimate the evolutionary response of CT_max_ in selection experiments, which is in close agreement with our findings of shallower latitudinal clines due to methodology (Santos et al. 2011). Selection with the “knockdown tube” (Huey et al. 1992) – an apparatus in which the temperature or time at which flies lose ability to cling on the walls of the tube and fall down can be readily recorded – has provided empirical evidence of the evolutionary potential of heat tolerance, resulting in an increase of nearly 2.5°C in knockdown temperature and a realized heritability of roughly 0.12 for this trait (Gilchrist and Huey 1999; see also McColl et al. 1996). Our results are in close agreement with these values, but suggest that the overall response to selection and heritability for the parameter CT_max_ may be even higher (although simulations are not meant to mimic the conditions of the knockdown tube, a decrease in average heat tolerance is expected simply due to stochasticity).

This theoretical framework can also explain why flies successfully selected for increasing knockdown resistance in the knockdown tube do not show a higher knockdown resistance than their respective controls when assayed in glass vials (Hoffmann et al. 1997). Although this discrepancy suggested to some researchers that the physiological and genetic mechanisms accounting for heat tolerance vary according to the methodology employed (Hoffmann et al. 1997, 2003b; Rako et al. 2007; Sgrò et al. 2010), we showed that the absence of correlation between heat tolerance indices is not evidence of different underlying mechanisms (Santos et al. 2011). Nonetheless, gender-specific patterns show that indices of physiological tolerance differ between methods: whereas, in the knockdown tube, *males are somewhat more resistant to knockdown than females* (Hoffmann et al. 1997; p. 394; see also Jenkins and Hoffmann 1994; Bubli et al. 1998), a common result in glass vials is that *D*. *melanogaster* females are more resistant to heat stress than males (Huey et al. 1991; Loeschcke et al. 1997; Mitchell and Hoffmann 2010; Parkash et al. 2010). These methodological differences (see also Folk et al. 2006) should be critically addressed before speculating about a putative independent genetic control of heat tolerance indices inferred from correlated responses (or lack thereof) across methodologies.

Second, our simulations show that the effects of stochasticity on mortality as temperatures increase do not have a major impact on the selection differential of CT_max_, hence genes for increased heat tolerance should be eventually selected regardless of the thermal regime. Thus, the main issue from an empirical perspective remains detecting, rather than eliciting, an evolutionary response (see also Santos et al. 2011). The prediction stemming from our results is that heat tolerance will increase to roughly the same level regardless the ramping rates employed during selection, which can be tested with the knockout tube or undertaking family selection experiments using the knockdown vial technique (which is advantageous because family means would be less affected by methodological noise, and selected flies would not be stressed). Similarly, should our results be extrapolated to natural conditions, they would suggest that daily and seasonal variations in the rate of change in temperature have only a minor effect on the overall evolutionary response in CT_max_, everything else being equal.

## Concluding remarks

This study arose from the paradox that the most widespread and common *Drosophila* species (Powell 1997) apparently exhibits limited adaptive potential for upper thermal limits (Mitchell and Hoffmann 2010). This conclusion is in striking conflict with the invasive success of *D*. *melanogaster* (which originated in sub-Saharan Africa and established in Europe and Asia, more recently, in both the New World and Australia), a species subjected to spatially varying selection for many traits, including thermotolerance (e.g., David and Capy 1988; Sezgin et al. 2004; Schmidt et al. 2005; Hoffmann and Weeks 2007; González et al. 2010). Here, we demonstrate that this contradiction may stem from the confusion between parameter (CT_max_) and estimator (knockdown temperature), with the unfortunate result that “ecologically realistic” assays yield highly downwardly biased estimates of upper thermal limits (Rezende et al. 2011; Santos et al. 2011) and their true evolutionary potential. A reviewer of Santos et al.'s (2011) paper considered the take-home message that estimates of CT_max_ are highly sensitive to methodology as rather depressing. Ironically, this message turns out to be good news because adaptive genetic responses for increasing upper thermal limits may be higher than acknowledged in recent studies.

Our results also illustrate how *the experimental approaches adopted might substantially affect the conclusions drawn from a particular investigation* (Chown et al. 2009; p. 138). Importantly, the solution to this problem does not entail compiling heat tolerance estimates under a myriad of conditions and increasingly intricate experiments (e.g., Terblanche et al. 2008; Chidawanyika and Terblanche 2011; Overgaard et al. 2011a). It is currently clear that the uncontrolled effects of cumulative thermal stress, its impact on correlated traits and on intrinsic survival probabilities may have a larger impact on heat tolerance estimates than the factors under study (Rezende et al. 2011; Santos et al. 2011). Many of the indices employed in recent studies are hardly comparable across systems, their precision and validity have not been assessed experimentally (repeatability estimates of measurements of thermal tolerance and other physiological limits are virtually absent; Santos et al. 2011; Krebs and Loeschcke 1997; see also Wolak et al. 2011; Castañeda et al. 2012) and neither has their “ecological relevance” (Rezende and Santos 2012). Therefore, resulting patterns should be carefully assessed in the light of our findings, which clearly show that there is substantially more to thermal limits and their potential to respond to selection than meets the eye.

## Supporting Information

**Appendix S1.** Stochasticity effects in heat knockdown resistance assays.

**Appendix S2.** Numerical results from simulation model 1 assuming additive and equal allele effects for CT_max_.

**Appendix S3.** Genetic Variation for CT_max_ assuming unequal allele effects and nonadditivity.
