# Supplementary material for: Keeping pace with climate change: what is wrong with the evolutionary potential of upper thermal limits?
Source: Ecol Evol. 2012 Oct 13;2(11):2866–80. doi: 10.1002/ece3.385 (PMC3501637; doi:10.1002/ece3.385)
Supplement: Supplementary file 1 [file ece30002-2866-SD1.pdf]

Supplementary Information for:

Keeping pace with climate change: what is wrong with the evolutionary potential of upper thermal limits?

Mauro Santos*, Luis E. Castañeda, and Enrico L. Rezende

* To whom correspondence should be addressed. E-mail: mauro.santos@uab.es.

Contents

Appendix S1: Stochasticity effects in heat knockdown resistance assays

Appendix S2: Numerical results from simulation model 1 assuming additive and equal allele effects for CT_{max}

Appendix S3: Genetic Variation for CT_{max} assuming unequal allele effects and nonadditivity

References

Appendix S1: Stochasticity effects in heat knockdown resistance assays

Heat tolerance was measured in twelve genetically identical individuals from each of ten independent isogenic lines of *Drosophila subobscura*: five with chromosome arrangement O_{3+4} and five with O_{st} (see Dolgova et al. 2010). Knockdown temperature was scored as the temperature for individual flies to be knocked down and immobilized in a dynamic assay, where temperature was increased at a constant rate until the end point was observed. We used two different heating rates: a slow ramping protocol with T_0 = 24 ºC and ΔT = 0.1 ºC min^{-1}, and a fast ramping assay with T_0 = 24 ºC and ΔT = 0.6 ºC min^{-1}.

The average knockdown temperature with slow ramping ($\text{mean} \pm \text{SD}:\ 34.22 \pm 1.81$ ºC) was substantially lower than with fast ramping (37.49 ± 0.83 ºC). This agrees with the results in *D. melanogaster* (Chown et al. 2009) and is expected from our theoretical treatment of ramping effects on heat tolerance (Rezende et al. 2011; Santos et al. 2011). The point here is that the proportion of survivors in any isogenic line gradually decreased from one to zero (Figure S1), indicating that stochasticity effects are inherent to thermotolerance assays (Santos et al. 2011). The within-line variance for knockdown temperature was significantly higher with slow ramping $\left(\hat{\sigma}^2_{\mathrm{slow}} = 2.1756\right)$ than with fast ramping $\left(\hat{\sigma}^2_{\mathrm{fast}} = 0.7768\right)$ ($F_{109,106} = 2.80,\ P < 0.001$), which is also indicative that stochasticity increased with time under heat stress.

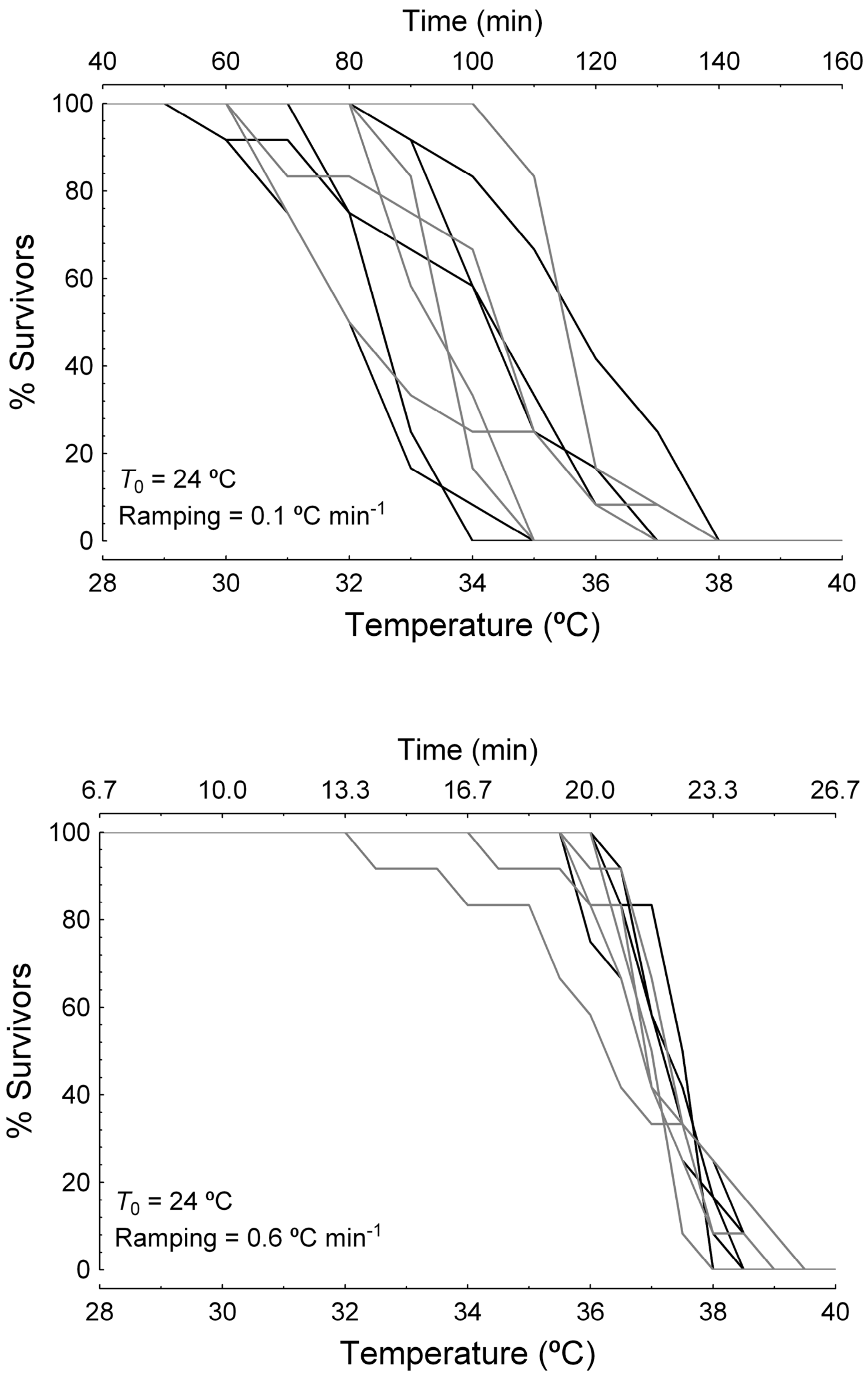

Figure S1: Each line represents the survivorship curve of up to twelve genetically identical *D. subobscura* flies from five isogenic lines with chromosome arrangement O_{3+4} (black lines), and five lines with chromosome arrangement O_{st} (grey lines). Upper panel: slow ramping. Lower panel: fast ramping.

Appendix S2: Numerical results from simulation model 1 assuming additive and equal allele effects for $\mathrm{CT_{max}}$

The genetic variable upper critical thermal limit ($\mathrm{CT_{max}}$) was assumed to be determined by $\ell = 20, 40$ autosomal diallelic loci with purely additive effects, and recombination frequencies $r = 0.05, 0.15, 0.25$ in females. For each combination of ℓ and r the same initial base population of $N = 5{,}000$ flies was subjected to 12 generations of up-selection for knockdown resistance (the top 20% of each sex was retained) under 400 different ramping protocols: initial temperatures T_0 ranged from 15 ºC to 34 ºC with interval 1 ºC, and ramping rates ΔT ranged from 0.05 ºC min^{-1} to 1 ºC min^{-1} with interval 0.05 ºC min^{-1}. All simulations assumed an average fruit fly weighing 1 mg with constant metabolic rate equal to 4.2 mL O_2 g^{-1} h^{-1} at 18 ºC. Its total energy budget prior to the heat knockdown assays was equal to 171.6 μL O_2, and $Q_{10} = 3.5$ (Santos et al. 2011).

Figures S2A – S2F: 3-D plots of (*i*) the realized heritabilities for knockdown temperature (kt), (*ii*) the increase of knockdown temperature after selection and (*iii*) the increase of $\mathrm{CT_{max}}$ after selection, against T_0 and ΔT. The realized heritability of knockdown temperature was estimated by regressing the response to selection against the cumulated selection differential over the 12 generations of selection. The plots for $\mathrm{CT_{max}}$ are framed in shadow because selection responses for this underlying physiological variable are hidden to the experimentalist.

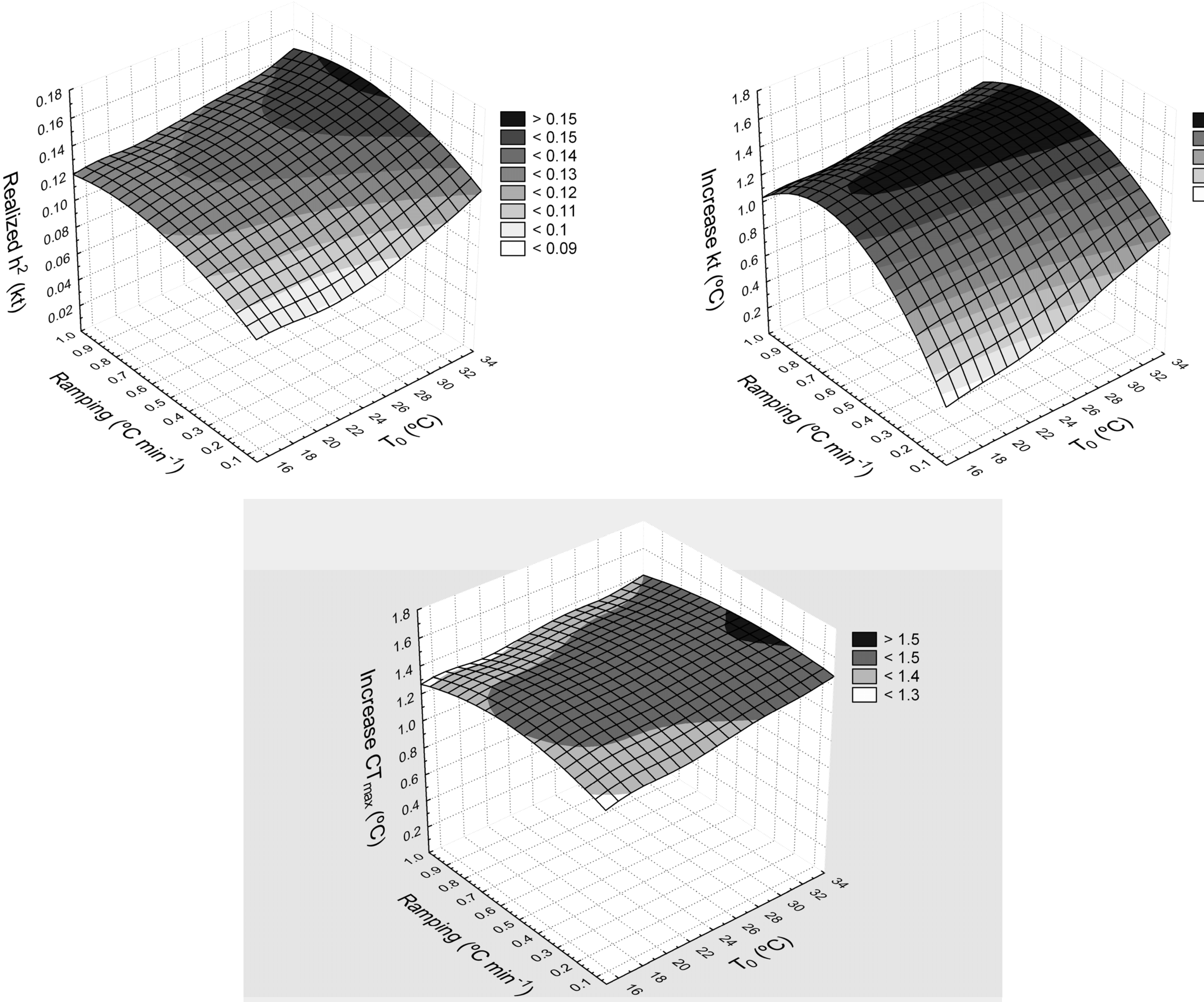

Fig. S2A: Simulation model 1. Census size N = 5,000 flies subjected to 12 generations of up-selection (top 20%) for knockdown temperature under 400 different ramping protocols. Loci: 20 for CT_{max}. Recombination frequency: r = 0.05.

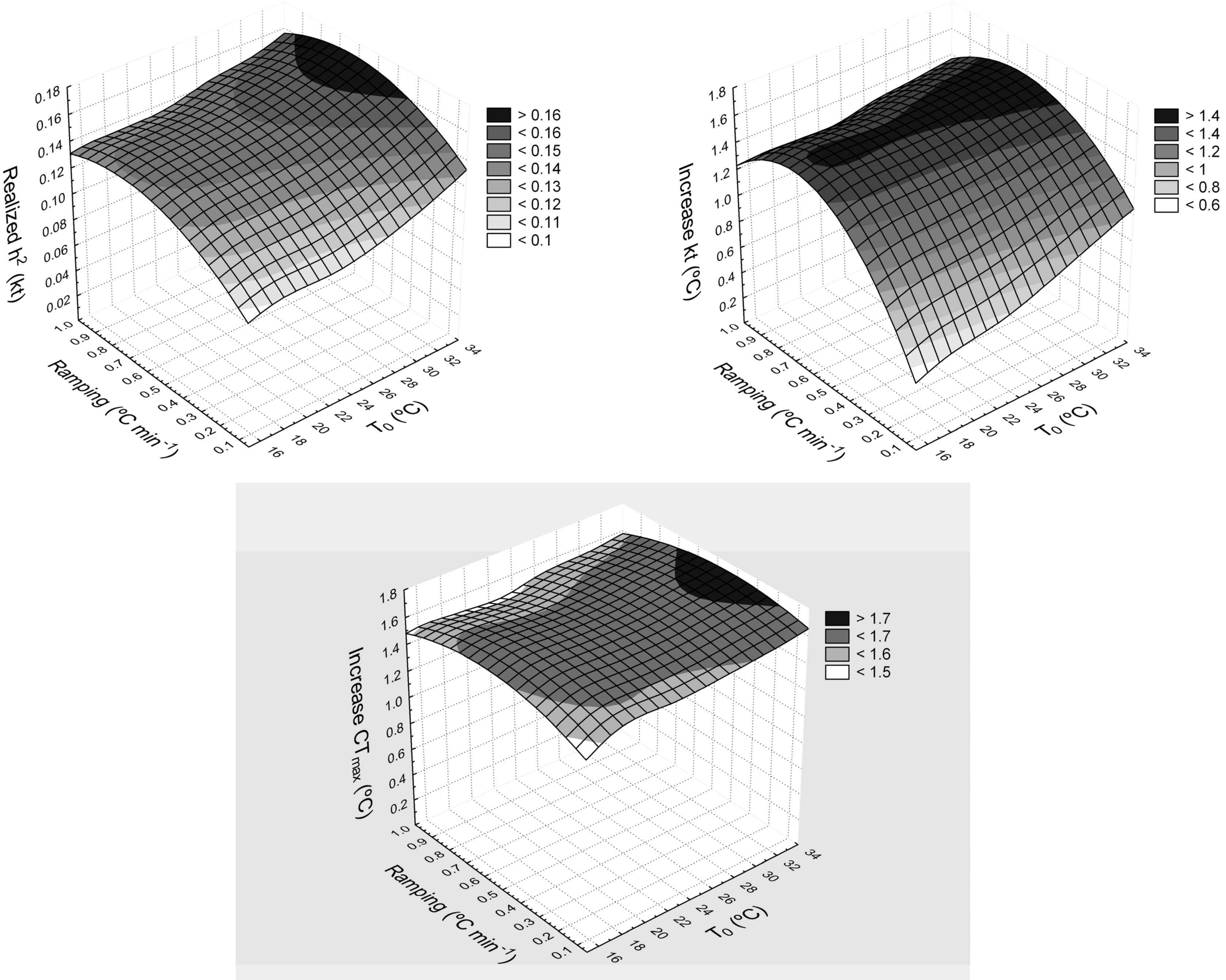

Fig. S2B: Simulation model 1. Same as fig. S2A with recombination frequency: $r = 0.15$.

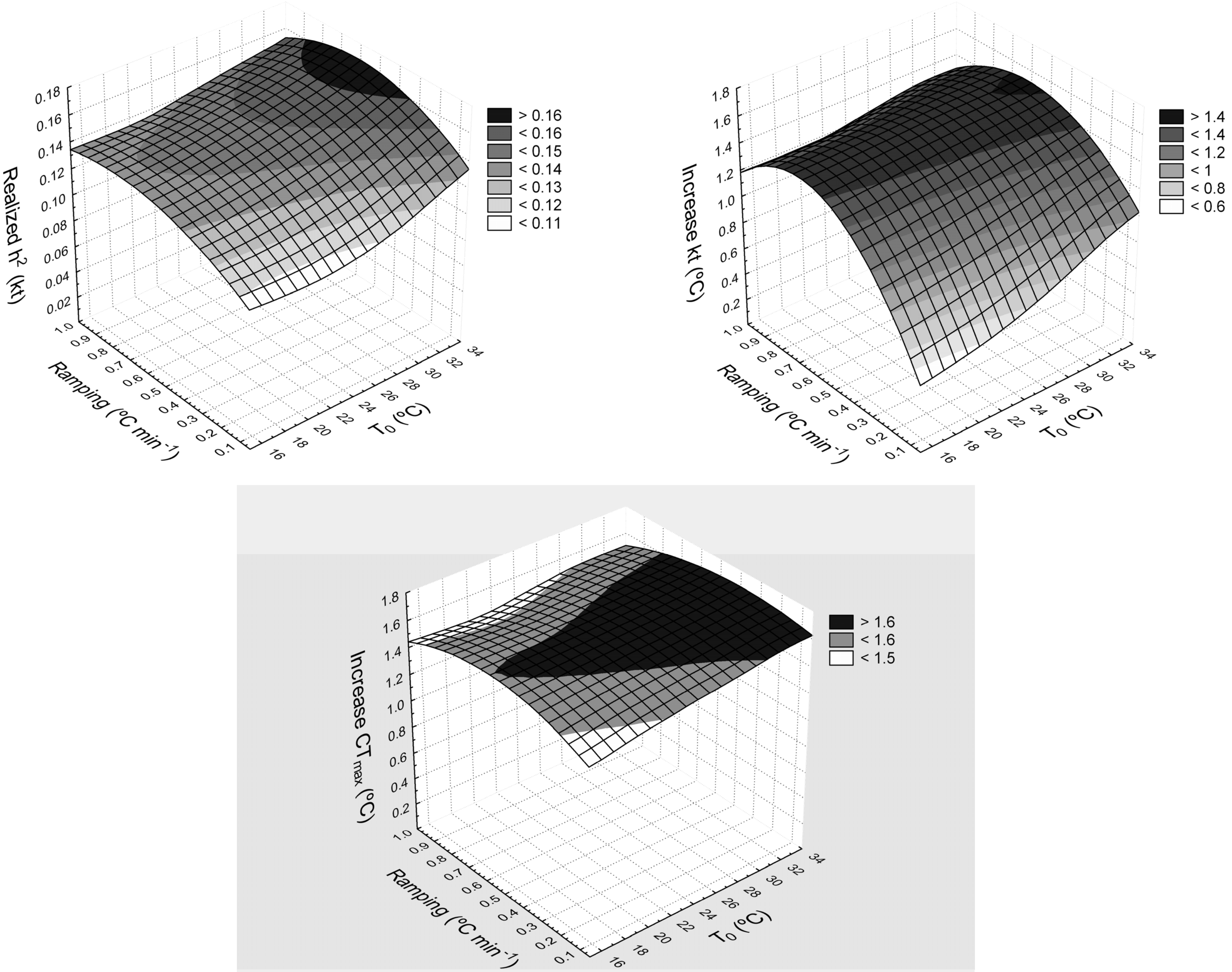

Fig. S2C: Simulation model 1. Same as fig. S2A with recombination frequency: $r = 0.25$.

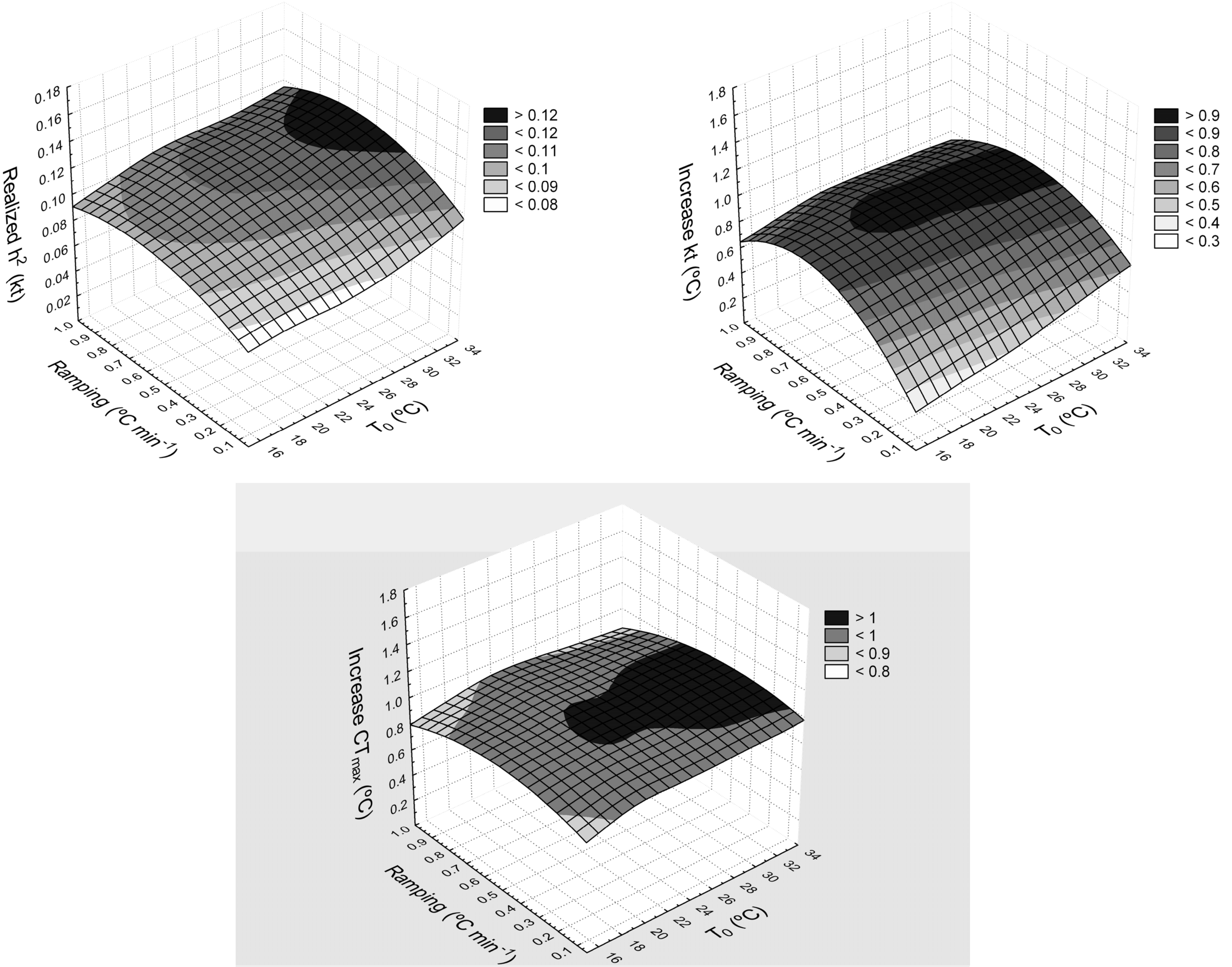

Fig. S2D: Simulation model 1. Census size N = 5,000 flies subjected to 12 generations of up-selection (top 20%) for knockdown temperature under 400 different ramping protocols. Loci: 40 for CT_{max}. Recombination frequency: r = 0.05.

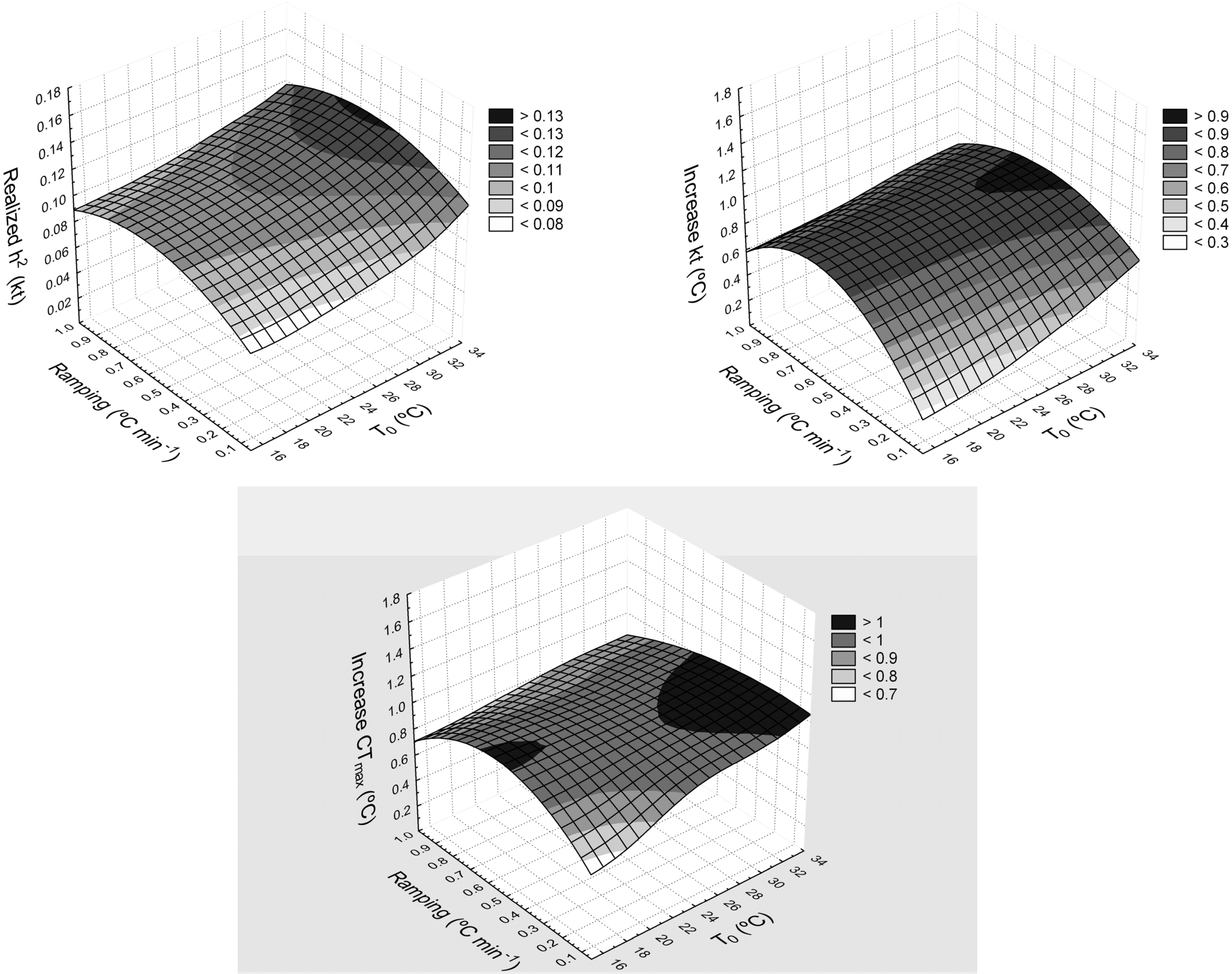

Fig. S2E: Simulation model 1. Same as fig. S2D with recombination frequency: $r = 0.15$.

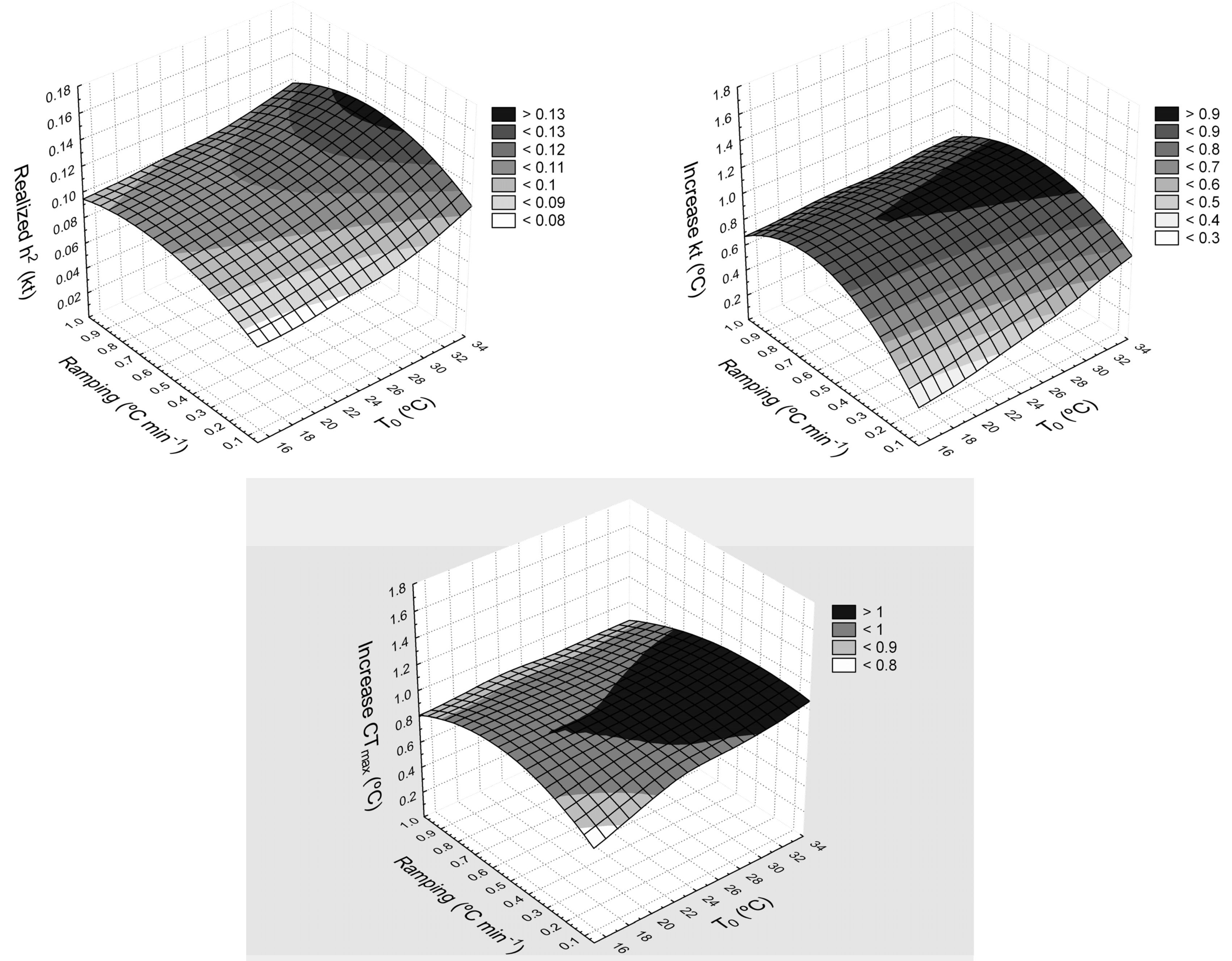

Fig. S2F: Simulation model 1. Same as fig. S2D with recombination frequency: $r = 0.25$.

Appendix S3: Genetic Variation for $\mathrm{CT}_{\max}$ assuming unequal allele effects and nonadditivity

To expand results from model 1 relaxing the assumptions of equal effects and strict additivity, we have also modeled an unequal distribution of allele effects, a_i, on $\mathrm{CT}_{\max}$ that vary in their degree of dominance, h_i. The genotypic values of genotypes with 0, 1 and 2 alleles ′1′ at the *i*th locus were 0, $h_i a_i$ and $2a_i$, respectively, with $1 \le h_i < 2$ ($h_i = 1$ reflects perfect additivity and $h_i = 2$ complete dominance of allele ′1′). The distribution of allele effects was obtained from a gamma distribution with shape parameter one-half and scale parameter one, which gives a coefficient of kurtosis of 12 and might realistically cover the actual distribution of allele effects on the trait (the greater the kurtosis, the more variance is contributed by a 'large-effect' gene). Our ignorance of the dominance relationships of alleles that affect $\mathrm{CT}_{\max}$ makes it difficult to model h_i. However, under the reasonable assumption that heat tolerance is correlated with fitness we would expect that 'deleterious' alleles (i.e., alleles ′0′ that do not increase heat tolerance) in loci with large effects on $\mathrm{CT}_{\max}$ tend to be recessive, whereas loci with small effects tend to be additive. This is because the distribution of coefficients of dominance for fitness is such that as mutant effects increase there is a tendency toward recessivity in the fitness scale, whereas mildly deleterious alleles tend to be roughly additive (Simmons and Crow 1977; see also Crow 2008). Therefore, we assumed an exponential function to define limits on the coefficient of dominance such that $h_i = 2 - \exp(-\beta a_i)$, where β (assumed equal to one; see Caballero and Keightley 1994) gives the slope of the function. Thus, for $a_i = 0$ the degree of dominance is $h_i = 1$ and as a_i increases (the maximum possible value is 2 ºC given that $\mathrm{CT}_{\max}$ was bounded between 38 ºC and 42 ºC; see above) the degree of dominance also increases up to a maximum of $h_i = 1.86$.

The loci controlling $\mathrm{CT}_{\max}$ were assumed to be on the same chromosome for simplicity, and the recombination fraction between adjacent loci was zero in males (as it happens in *Drosophila*) and r in females with no interference. The recombination process followed the stochastic multilocus method described in Fraser and Burnell (1970). All procedures

to select for increasing knockdown temperature were the same as in model 1 (see text). Simulations also assumed an average fruit fly weighing 1 mg with constant MR of $4.2\ \text{mL O}_2\ \text{g}^{-1}\ \text{h}^{-1}$ at 18 ºC (0.07 μL O_2 per fly min), with a total energy budget before the heat knockdown assay equal to 171.6 μL O_2 and $Q_{10} = 3.5$.

RESULTS

Results from sample simulations with $\ell = 20$ and $r = 0.25$ are plotted in Fig. S3A-D (cf. with Fig. 1A-D in the manuscript) for flies selected with a fast ramping assay ($T_0 = 28$ ºC, ΔT = 0.5 ºC min^{-1}), and in Fig. S3E-H (cf. with Fig. 2A-D in the manuscript) for the same flies selected with a slow ramping protocol ($T_0 = 28$ ºC, ΔT = 0.06 ºC min^{-1}). In these simulations the initial genotypic variance V_G was 0.31 $(ºC)^2$ (Fig. S3D, S3H) and substantially higher than in the additive and equal allele effects situation [~ 0.08 $(ºC)^2$; see Fig. 1D, 2D in the manuscript], with a 'major' gene that quickly shifted to fixation (Fig. S3C, S3G) initially contributing 63% to V_G.

With fast ramping the realized heritability was 0.140 (Fig. S3A), but it dropped to 0.100 with slow ramping (Fig. S3E). However, the response of CT_{max} to directional selection on knockdown temperature was again essentially independent of the ramping conditions (ΔCT_{max} = 2.45 ºC with fast ramping and ΔCT_{max} = 2.43 ºC with slow ramping; Fig. S3B, S3F), and the response of knockdown temperatures substantially lower with slow ramping (Δkt_{fast} = 2.1 ºC *vs*. Δkt_{slow} = 1.1 ºC; Fig. S3A, S3E). Therefore, the results were not significantly different to those obtained assuming additive and equal allele effects. Extensive computer simulations as those performed in Appendix S2 reinforce this conclusion (results not shown).

Figure S3A-D: Sample numerical results from simulation model 1 assuming unequal allele effects with nonadditive contributions to $\mathrm{CT_{max}}$. The census size at each generation was $N = 5{,}000$ flies. They were subjected to 50 generations of up-selection for knockdown temperature (the top 20% of each sex was retained) using a fast ramping protocol with $T_0 = 28$ ºC and $\Delta T = 0.5$ ºC min^{-1}. $\mathrm{CT_{max}}$ was controlled by 20 diallelic loci on the same chromosome, with recombination frequency between adjacent loci $r = 0.25$ in females. Allelic frequencies in the base population ranged from $p = 0.1$ to $p = 0.5$ for alleles $'1'$, with additive effects ranging from $a_i = 1.1\times10^{-5}$ ºC to $a_i = 0.56$ ºC and dominance relationships from $h_i = 1$ to $h_i = 1.43$. Heritability of $\mathrm{CT_{max}}$ was $h^2 = 0.25$ in the base population. Panel A plots the increase in knockdown temperature and its realized heritability, estimated by regressing the response to selection against the cumulated selection differential over the first 7 generations of selection when all loci still remained polymorphic (5% criterion). Panel B plots the increase in $\mathrm{CT_{max}}$, which was 17% higher relative to the increase in knockdown temperature. Panel C plots the frequency changes of alleles $'1'$ increasing $\mathrm{CT_{max}}$; the first allele to approach fixation was that for the 'major' gene with additive effect $a_i = 0.56$ ºC. Panel D plots the total genotypic variance V_G together with its causal components. V_G was decomposed into additive (V_A), dominance (V_D), and linkage disequilibrium components (D_L; the variance $D_{\mathrm{H\text{-}W}}$ due to deviations from Hardy-Weinberg equilibrium was assumed to be zero). Plots for $\mathrm{CT_{max}}$, allele frequencies and variance components are framed in shadow because their responses to selection for knockdown temperature are hidden to the experimentalist.

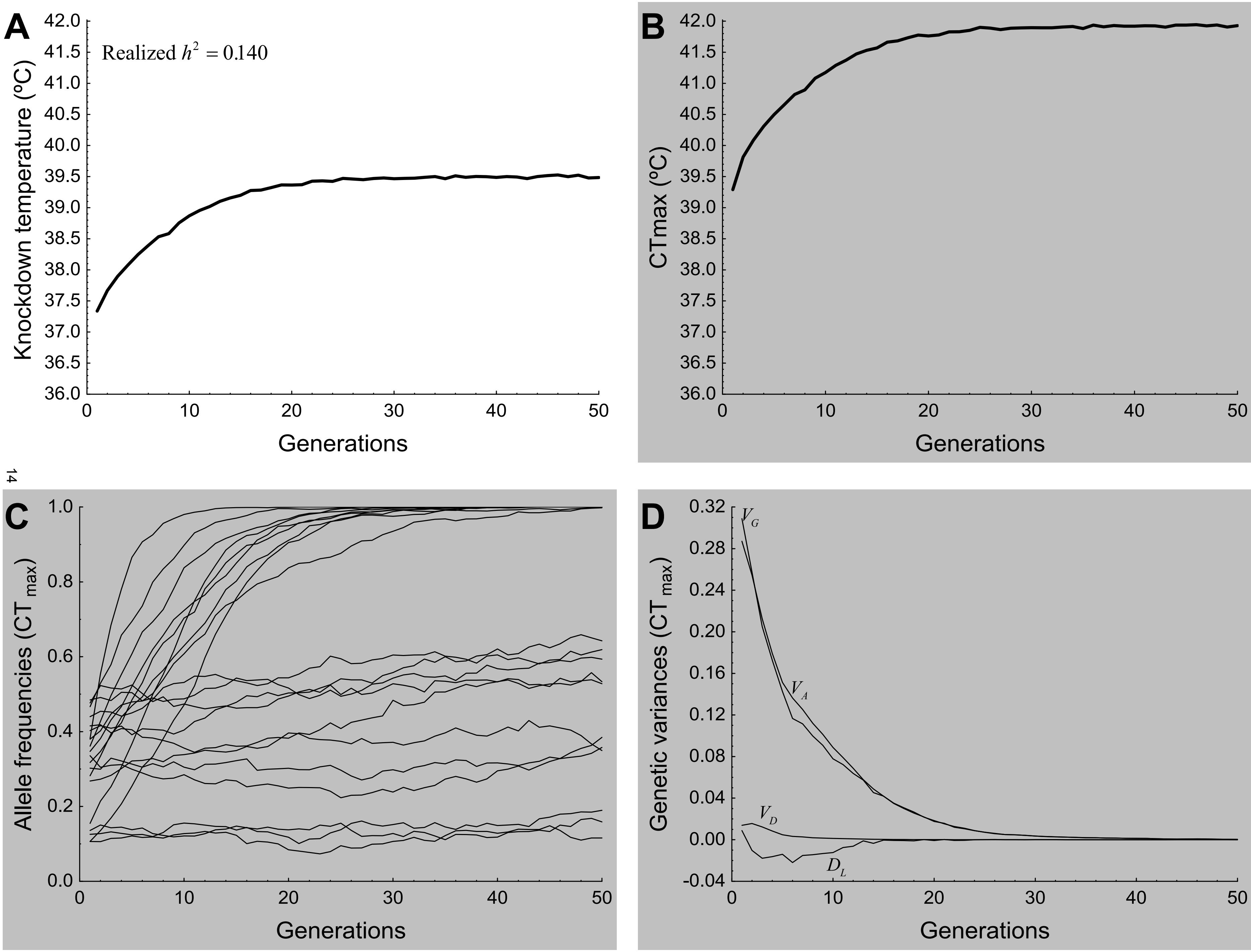

A
Realized $h^2 = 0.140$
Knockdown temperature (ºC)
Generations
B
CTmax (ºC)
Generations
C
Allele frequencies (CT_{max})
Generations
D
Genetic variances (CT_{max})
V_G
V_A
V_D
D_L
Generations

Figure S3E-H: Sample numerical results from simulation model 1 assuming unequal allele effects with nonadditive contributions to $\mathrm{CT_{max}}$. The census size at each generation was $N = 5{,}000$ flies. They were subjected to 50 generations of up-selection for knockdown temperature (the top 20% of each sex was retained) using a slow ramping protocol with $T_0 = 28$ ºC and ΔT = 0.06 ºC min^{-1}. $\mathrm{CT_{max}}$ was controlled by 20 diallelic loci on the same chromosome, with recombination frequency between adjacent loci $r = 0.25$ in females. Allelic frequencies in the base population ranged from $p = 0.1$ to $p = 0.5$ for alleles ′1′, with additive effects ranging from $a_i = 1.1\times10^{-5}$ ºC to $a_i = 0.56$ ºC and dominance relationships from $h_i = 1$ to $h_i = 1.43$. Heritability of $\mathrm{CT_{max}}$ was $h^2 = 0.25$ in the base population. Panel E plots the increase in knockdown temperature and its realized heritability, estimated by regressing the response to selection against the cumulated selection differential over the first 7 generations of selection when all loci still remained polymorphic (5% criterion). Panel F plots the increase in $\mathrm{CT_{max}}$, which was 2.2 times higher than the increase in knockdown temperature. Panel G plots the frequency changes of alleles ′1′ increasing $\mathrm{CT_{max}}$. Panel H plots the total genotypic variance V_G together with its causal components. V_G was decomposed into additive (V_A), dominance (V_D), and linkage disequilibrium components (D_L; the variance $D_{\mathrm{H\text{-}W}}$ due to deviations from Hardy-Weinberg equilibrium was assumed to be zero). Plots for $\mathrm{CT_{max}}$, allele frequencies and variance components are framed in shadow because their responses to selection for knockdown temperature are hidden to the experimentalist.

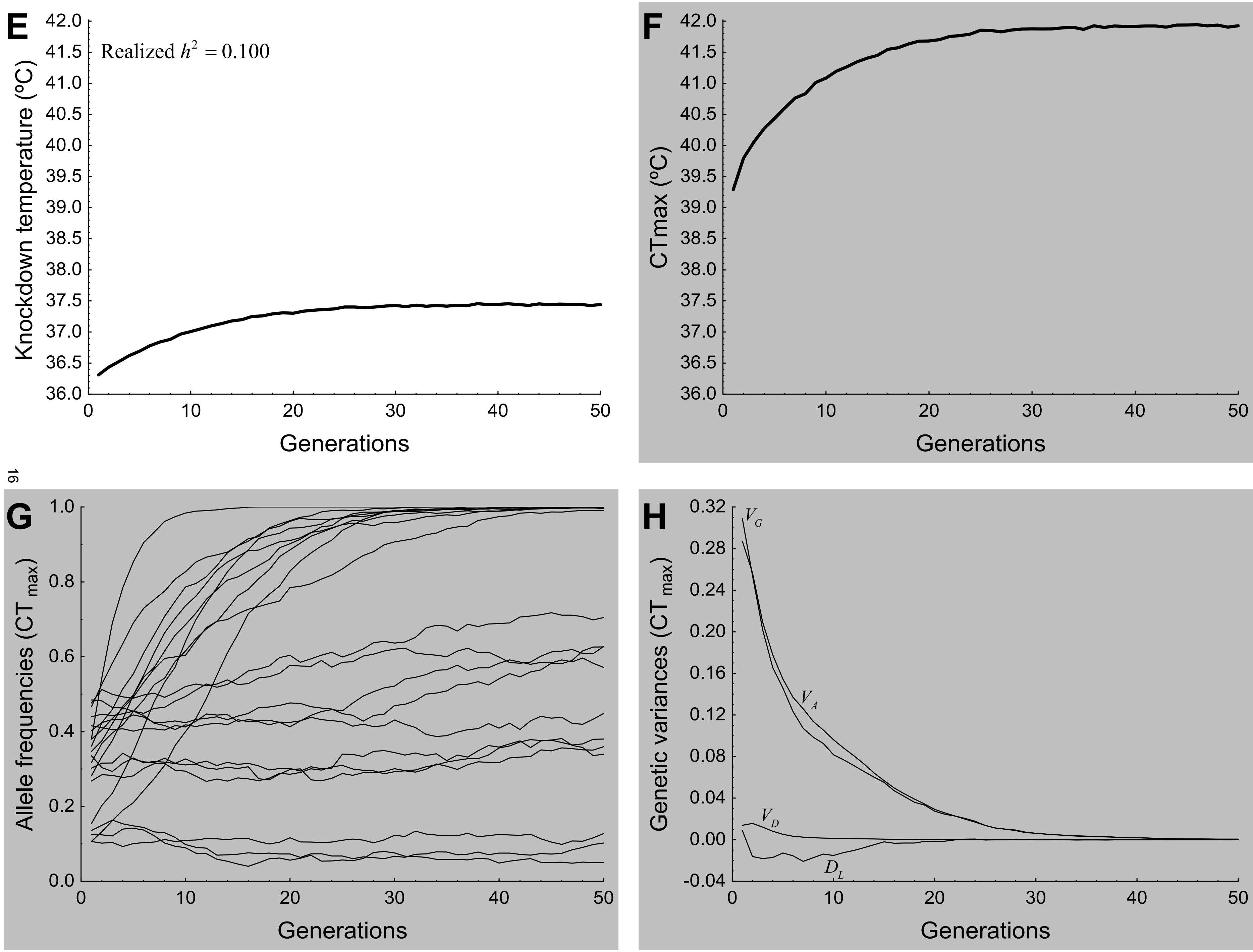
E
Realized $h^2 = 0.100$
Knockdown temperature (ºC)
Generations
F
CTmax (ºC)
Generations
G
Allele frequencies (CT max)
Generations
H
Genetic variances (CT max)
V_G
V_A
V_D
D_L
Generations

References

Caballero, A., and P. D. Keightley. 1994. A pleiotropic nonadditive model of variation in quantitative traits. *Genetics* **138**:883-900.

Chown, S. L., K. R. Jumbam, J. G. Sørensen, and J. S. Terblanche. 2009. Phenotypic variance, plasticity and heritability estimates of critical thermal limits depend on methodological context. *Funct. Ecol.* **23**:133-140.

Crow, J. F. 2008. Maintaining evolvability. *J. Genet.* 87:349-353.

Dolgova, O., C. Rego, G. Calabria, J. Balanyà, M. Pascual, E. L. Rezende, and M. Santos. 2010. Genetic constraints for thermal coadaptation in *Drosophila subobscura*. *BMC Evol. Biol.* **10**:363.

Fraser, A., and D. Burnell. 1970. Computer models in genetics. McGraw-Hill, New York.

Rezende, E. L., M. Tejedo, and M. Santos. 2011. Estimating the adaptive potential of critical thermal limits: methodological problems and evolutionary implications. *Funct. Ecol.* **25**:111-121.

Santos, M., L. E. Castañeda, and E. L. Rezende. 2011. Making sense of heat tolerance estimates in ectotherms: lessons from *Drosophila*. *Funct. Ecol.* **25**:1169-1180.

Simmons, M. J., and J. F. Crow. 1977. Mutations affecting fitness in *Drosophila* populations. *Ann. Rev. Genet.* **11**:49-78.
