# Supplementary figures and images for: Keeping pace with climate change: what is wrong with the evolutionary potential of upper thermal limits?
Source: Ecol Evol. 2012 Oct 13;2(11):2866–80. doi: 10.1002/ece3.385 (PMC3501637; doi:10.1002/ece3.385)

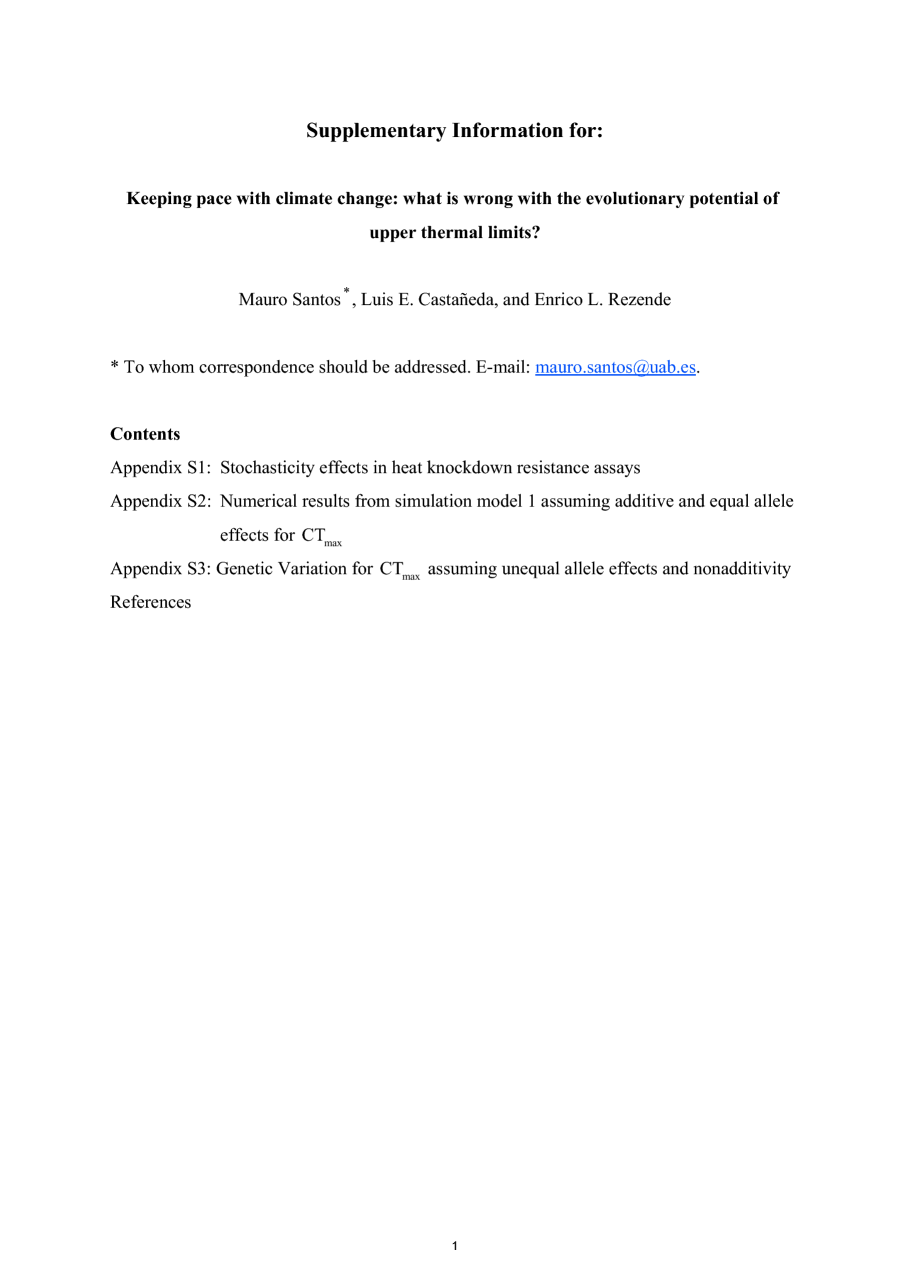

Supplement: Supplementary file 2 [file ece30002-2866-SD4.png]
